# Multi-Omics Meets Premalignancy: Paving the Way for Early Prevention of Cancer

**DOI:** 10.34133/research.0930

**Published:** 2025-10-17

**Authors:** Feiran Zhang, Ziyi Zhou, Peng Zhang, Shao Li

**Affiliations:** Institute for TCM-X, MOE Key Laboratory of Bioinformatics, Bioinformatics Division, BNRist, Department of Automation, Tsinghua University, Beijing 100084, China.

## Abstract

Comprehensive understanding of premalignant lesions (PMLs) represents a pivotal opportunity for cancer early detection and interception. Recently, advances in multi-omics technologies and artificial intelligence (AI) methods have provided unprecedented insights into PML-induced tumorigenesis. In this paper, we firstly catalog clinically recognized PMLs across 15 cancer types, emphasizing their epidemiological profiles and malignant transformation potentials. Then, we summarize recent intriguing discoveries and remaining challenges from bulk, single-cell, and spatial omics studies, highlighting how these omics technologies reveal the dynamic molecular, cellular, and spatial evolution from precancerous states to invasive malignancies. We further discuss network-based computational strategies for multi-omics integration and tumorigenesis trajectory inference, with applications of recent deep learning-based AI approaches. Finally, we highlight translational implications for PMLs, including developing high-precision early-diagnosis biomarkers and targeted pharmacological preventive strategies. Collectively, this paper underscores how the convergence of high-resolution multi-omics with sophisticated AI is poised to redefine PML research, enabling pan-cancer exceedingly-early risk stratification and pharmacological prevention.

## Introduction

Premalignant lesions (PMLs) are diseases with the potential to progress to cancer and, thus, are considered precursor states and marked risk factors of malignancy in clinical practice. Tumorigenesis associated with PMLs manifests across most human organs, encompassing a characteristic multistep progression from normal tissue to cancer, which can pathologically be categorized into (a) healthy tissue, (b) chronic inflammation, (c) hyperplasia and/or metaplasia, (d) low-grade dysplasia/intraepithelial neoplasia (LGD/LGIN), (e) high-grade dysplasia/intraepithelial neoplasia (HGD/HGIN), or carcinoma in situ (CIS), and (f) invasive cancer [1]. This prolonged process of tumorigenesis, starting from intrinsic or environmental factors, often lasts clinically for more than 10 years, comprising step-wise accumulation of driver mutations and acquisition of cancer hallmark capabilities [2,3]. Investigation of PMLs and their evolutionary trajectories offers critical insights into the underlying biological mechanisms governing cancer initiation, aiding in accurate tumor risk stratification, and targeted and personalized intervention prior to cancer onset in clinic practice [4].

The evolution from PMLs to invasive carcinoma is a long-term, rare, and mechanistically complex dynamic process. Investigating the biological mechanisms of PMLs requires examination across distinct dimensions, including molecular alterations, cellular heterogeneity, and spatial tissue organization. Understanding this progression from a multi-level and dynamic perspective is both a research focus and a critical challenge in the field. With the advancement of sequencing technologies, particularly the maturation of single-cell multi-omics and spatial sequencing techniques, we have gained systematic insights and novel perspectives on homeostatic and tumor-associated cellular landscapes and their spatial distributions across multiple human organs [5,6]. In this regard, researchers have begun applying multi-omics technologies to decipher the biological underpinnings of PMLs across diverse organs, leading to a wealth of discoveries [7–12], and initiatives such as the PreCancer Atlas and the Human Tumor Atlas Network (HTAN) [13,14]; in parallel, employing systems biology and artificial intelligence (AI) methodologies to analyze multi-omics data pertaining to premalignant-to-cancer transitions and elucidate dynamic tumorigenesis patterns is emerging as a prominent research focus within this domain. Although several reviews have reviewed PMLs and tumorigenesis mechanisms [3,4,15], there is a lack of a comprehensive review addressing PML research through the lens of multi-omics and systematic analysis, representing a gap recognized by investigators in this field.

To this end, we have conducted a systematic compilation of multi-omics studies concerning PMLs across diverse organs and present this comprehensive review. Building upon a systematic exposition of PML disease entities, this review delineates recent advancements in multi-omics research, encompassing bulk, single-cell, and spatial omics approaches, as well as the key challenges remaining to be addressed. Subsequently, we present an overview of representative systems biology methodologies employed for integrative and dynamic analysis of multi-omics data within the PML context. Furthermore, we examine the clinical translational implications of these investigations, in the domains of cancer risk stratification and early intervention strategies. Finally, we highlight several promising research directions centered on emerging multi-omics sequencing methods, analytical frameworks, and clinical incorporation for PML investigation. We anticipate that this review will serve as a systematic reference for researchers in this domain by compiling recent advancements in PML-related multi-omics research, while also offering novel perspectives on leveraging multi-omics technologies and computational approaches to elucidate the biological foundations of premalignant states and develop innovative cancer prevention strategies.

## Pathological Catalogue and Epidemiology of PMLs

To systematically review and categorize PMLs across diverse organs, here we summarize clinically established PMLs associated with 15 cancer types across 12 distinct organs, categorized according to the histological classification of their corresponding carcinomas: squamous cell carcinoma, adenocarcinoma, and other carcinoma types. A summary table with their relative risks or progression rates to malignancy is presented in Table 1. It is worth mentioning that PMLs are increasingly recognized as entities requiring definition through the integration of molecular characteristics with pathological morphological features [1]. This section focuses specifically on pathological and epidemiological dimensions, with molecular characteristics of PMLs addressed in subsequent sections.

**Table 1.** Catalogue of premalignant lesions

Organ	Cancer	Disease name	Relative risk (RR)/Progression rate	Reference
Breast	BRCA	Atypical ductal hyperplasia (ADH)	RR 3.9 (risk for both invasive and in situ BRCA)	[160]
		Atypical lobular hyperplasia (ALH)	RR 4.7 (risk for both invasive and in situ BRCA)	
		Ductal carcinoma in situ (DCIS)	25%–60% progress to invasive BRCA if untreated within 9–24 years	
		Lobular carcinoma in situ (LCIS)	RR 8–10	
Cervix	CESC	Cervical intraepithelial neoplasia 1–3 (CIN-1–3)	RR 3.30 (after treatment)	[19]
		Low-grade squamous intraepithelial lesion (LSIL)		
		High-grade squamous intraepithelial lesion (HSIL)		
Colorectum	COAD	Crohn’s disease (CD)	RR 1.66	[161–163]
		Ulcerative colitis (UC)	RR 1.4	
		Familial adenomatous polyposis (FAP)	RR 4.61 (after colectomy)	
		Serrated adenoma	RR 3.07 (4.76 with dysplasia)	
		Conventional adenoma	RR 2.51	
Esophagus	EAC	Barrett’s esophagus (BE)	RR 11.3	[164]
		BE with dysplasia	RR 4.8 (compared to BE without dysplasia)	
	ESSC	Esophagitis	RR 1.96	[165,166]
		Basal cell hyperplasia	RR 3.06	
		Esophageal squamous dysplasia (ESD)	RR 4.55 (mild), 15.18 (moderate)	
		Esophageal squamous carcinoma in situ	RR 55.78	
Liver	LIHC	Chronic hepatitis	RR 1.7 (all-cause mortality)	[25,167]
		Liver fibrosis	RR 5.8 (liver-related death)	
		Cirrhosis	RR 12.7 (liver-related death)	
		Hyperplastic nodule (HN)	RR 2.96 (low-grade),16.8 (high-grade), compared to HN	
		Dysplastic nodule (DN)		
Lung	LUSC	Lung squamous hyperplasia	NA	[168]
		Lung squamous metaplasia	∼9%	
		Lung squamous dysplasia	∼9% (mild/moderate dysplasia)	
		Lung squamous cell carcinoma in situ	50% progress to LUSC within 2 years	
	LUAD	Atypical adenomatous hyperplasia (AAH) of lung	NA	[23]
		Adenocarcinoma in situ (AIS) of lung	5.6% recurrence of lung cancer at 10 years	
Mouth	OSCC	Oral lichen planus	1.14% mean malignant transformation rate	[169–171]
		Oral mucosa leukoplakia	3.5% mean malignant transformation rate	
		Oral erythroplakia	26.3% mean malignant transformation rate	
		Oral submucous fibrosis	Overall 6% risk of malignant transformation	
Ovary	HGSOC	Serous tubal intraepithelial carcinoma (STIC)	Average 6.5 year for STIC progression to HGSOC	[172]
Pancreas	PDAC	Chronic pancreatitis (CP)	RR 6.9	[21,173]
		Pancreas acinar ductal metaplasia (ADM)	NA	
		Intraductal papillary mucinous neoplasm (IPMN)	15% progress to pancreatic malignancy in 15 years	
		Pancreatic intraepithelial neoplasia (PanIN)	1.3%–1.5% lifetime probability of progressing from PanIN 1 to PDAC	
		Mucinous cystic neoplasm (MCN)	NA	
Prostate	PRAD	Atrophy of prostate	NA	[174]
		Atypical adenomatous hyperplasia (AAH)	RR 2.06	
		Prostatic intraepithelial neoplasia (PIN)	RR 3.06 (low-grade), 6.16 (high-grade)	
Skin	cSCC	Actinic keratosis (AK)	RR 7.7 (developing cSCC)	[175,176]
		Bowen’s disease	3% and 5% progress to invasive SCC if untreated	
	UVM	Melanocytic nevus	RR 1.41 (1–2 nevi) and 2.49 (≥3 nevi)	[27,177]
		Dysplastic nevus, Clark’s nevus	RR 10.1	
Stomach	STAD	Chronic atrophic gastritis (CAG)	RR 4.5 (compared to normal)	[178]
		Intestinal metaplasia (IM)	RR 6.2 (compared to normal), 1.74 (compared to CAG)	
		Low-grade dysplasia (LGD)	RR 10.9 (compared to normal), 3.93 (compared to CAG)	
		High-grade dysplasia (HGD)	RR 40.1 (compared to CAG)	

Squamous cell carcinomas demonstrate well-established PMLs across 5 organs: lung, oral cavity, esophagus, skin, and cervix. Esophageal squamous cell carcinomas (ESCCs) exhibit a typical PML progression cascade encompassing hyperplasia, dysplasia, and CIS, whereas lung squamous cell carcinomas (LUSCs) manifest an additional columnar-to-squamous metaplasia phase after hyperplasia. In cutaneous squamous cell carcinoma (cSCC), actinic keratosis (AK) represents a prevalent precursor lesion, exhibiting a global prevalence of 14% and conferring a relative risk of 7.7 for cSCC development [16]. Oral squamous cell carcinoma (OSCC) demonstrates a spectrum of PMLs, with oral submucous fibrosis and leukoplakia representing the most prevalent entities, exhibiting global prevalence rates of 4.96% and 4.11%, respectively [17]; oral erythroplakia demonstrates the highest malignant transformation rate, with 26.3% of cases progressing to OSCC (Table 1). Cervical squamous cell carcinoma (CESC) is frequently preceded by cervical intraepithelial neoplasia (CIN) following HPV infection, exhibiting an incidence rate of 270 per 100,000 in the United States [18]. Although CIN treatment demonstrates high efficacy, women remain at an elevated risk of developing CESC thereafter, with a reported relative risk of 3.30 [19], underscoring the necessity for continuous surveillance.

Another major class of PMLs is derived from organs with adenocarcinomas, including esophagus, stomach, colon, pancreas, lung, breast, and prostate. For the 4 digestive organs, chronic inflammation frequently precedes metaplastic and dysplastic transformations, such as chronic atrophic gastritis (CAG) and chronic pancreatitis. As for metaplasia, Barrett’s esophagus (BE) and gastric intestinal metaplasia (GIM) have a shared intestinal phenotype, whereas the reverse process, gastric (pyloric) metaplasia, manifests in approximately 28% of inflammatory bowel disease (IBD) patients [20]. Notably, gastric metaplasia demonstrates association with serrated polyp-initiated colorectal cancer (CRC) [7], suggesting reciprocal metaplastic phenomena between upper and lower gastrointestinal tract. Pancreatic carcinogenesis involves acinar-to-ductal metaplasia (ADM) as a precursor to pancreatic intraepithelial neoplasia (PanIN) preceding pancreatic ductal adenocarcinoma (PDAC) development. Intraductal papillary mucinous neoplasm (IPMN) represents another established PDAC precursor, exhibiting an estimated population prevalence of 10.9% and demonstrating a 15% progression rate to PDAC over 15 years [21,22]. For CIS entities including adenocarcinoma in situ (AIS) and minimally invasive adenocarcinoma (MIA) of lung adenocarcinoma (LUAD), surgical resection constitutes standard therapeutic intervention, typically associated with favorable prognostic outcomes. Postoperative AIS/MIA patients demonstrate 5-year recurrence-free survival, with secondary primary lung cancer incidence rates of 5.6% and 7.7% at 10 years, respectively [23]. Ductal CIS (DCIS) demonstrates 5-year recurrence rates of 13.6% (1978 to 1998) and 6.6% (1999 to 2010) following breast-conserving surgery, partially attributable to advancements in screening modalities and increased utilization of radiation and endocrine therapies [24].

Beyond squamous cell carcinoma and adenocarcinoma, several cancer types also have established PMLs. Liver hepatocellular carcinoma (LIHC) pathogenesis involves established risk factors including hepatitis B/C viruses, alcohol, and metabolic dysfunction-associated steatotic liver disease, progressing through hepatitis, liver fibrosis, cirrhotic lesions, dysplastic nodule, and, ultimately, carcinomas [25]. High-grade serous carcinomas (HGSOCs), representing 70% to 80% of global ovarian cancer burden [26], are suggested to originate from fallopian tube fimbria through precursor lesions of serous tubal intraepithelial carcinomas (STICs). Despite insufficient pathological standardization, dysplastic nevus (DN, also known as Clark’s nevus) remains recognized as a robust and consistent melanoma risk factor, conferring a 10-fold elevated risk [27]. Conversely, malignant transformation of individual DN to melanoma is rare, with lifetime risk estimates of 0.03% for males and 0.009% for females [27]. In summary, while progression rates among individual PMLs demonstrate considerable variation, the majority of PMLs are associated with substantially elevated cancer development risks, emphasizing the need for enhanced understanding of underlying mechanisms, as well as precise risk stratification and effective clinical intervention.

## Multi-Omics Profiling of the PMLs

**Fig. 1.**
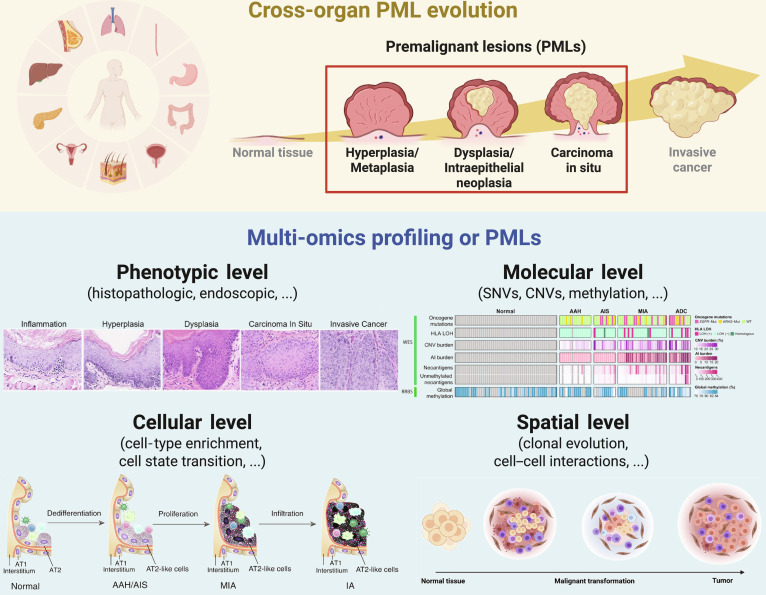
Overview of PMLs and paradigm of multi-omics studies. PMLs play an important role in the multistep process of cancer development, which typically involves a series of pathological changes from normal tissue to hyperplasia, metaplasia, dysplasia, and, ultimately, invasion. Through phenotypic, bulk, single-cell, and spatial omics analyses of recognized PMLs, we can identify the features or patterns of premalignancy development and progression. Elements in the “Phenotypic level”, “Molecular level”, “Cellular level”, and “Spatial level” panels are adapted from Yao et al. [156], Dejima et al. [157], Wang et al. [62], and Chen et al. [158], respectively. Figure created with Biorender.com. PMLs, premalignant lesions.

The advancement of multi-omics technologies, particularly single-cell sequencing and spatial transcriptomics, has provided new perspectives on the biological basis and dynamic evolution of PMLs, and is now becoming the leading paradigm in PML research. In this section, we systematically review studies of PMLs where different omics technologies were utilized and summarize the latest advances in multi-omics research across various organs from 3 perspectives: bulk level, single-cell level, and spatial level (Fig. 1 and Table 2).

**Table 2.** Representative omics-driven studies of premalignant lesions

Organ	Omics	Lesions	Year	Summary	Reference
Breast	Single-cell DNA (scDNA)	DCIS, BRCA	2018	Topographic single-cell sequencing data revealed a direct genomic lineage between in situ and invasive tumor subpopulations and further showed that most mutations and copy number aberrations evolve within the ducts prior to invasion.	[179]
	Spatial proteomics (SP)	DCIS, BRCA	2021	This comprehensive study within the HTAN Breast PreCancer Atlas offered insight into the etiologies of DCIS, its transition to IBC, and emphasized the importance of the tumor microenvironment (TME) stroma in promoting these processes.	[8]
	Single-cell RNA (scRNA)	DCIS	2022	Single-cell transcriptomic profiling of DCIS and invasive ductal carcinoma (IDC) provided novel insight into the intratumoral heterogeneity and molecular features of DCIS, which exhibit properties similar to IDC.	[180]
Cervix	scRNA	CIN, CESC	2022	This study was the first to delineate the transcriptome profile of immune cells during CESC progression using scRNA sequencing, and had implications for the development of novel treatments to inhibit the initiation and progression of CESC.	[181]
Esophagus	DNA	BE, EAC	2014	In conclusion, mutations in EAC driver genes generally occur exceptionally early in disease development with profound implications for diagnostic and therapeutic strategies.	[182]
	scRNA	Mutagen-treated mouse	2020	A mouse model of ESCC was created. The role of interplay between carcinogen-transformed epithelial cells and their microenvironment in ESCC development was reported, and a set of key transitional signatures associated with oncogenic evolution of epithelial cells were identified.	[156]
	Single-cell assay for transposase-accessible chromatin (scATAC)	BE	2021	Barrett’s esophagus arises on a gastric cis-regulatory landscape, characterized by single-cell-level co-activation of gastric and intestinal enhancers, mutational patterns reflecting a stomach-derived origin, and extensive intra-gland phenotypic heterogeneity that argues against strict stem-cell governance.	[58]
	scRNA, assay for transposase-accessible chromatin (ATAC), methylation (MET), DNA	BE	2021	A single-cell approach to investigate the cell of origin for Barrett’s esophagus (BE) and the mechanisms leading to the development of esophageal adenocarcinoma (EAC) revealed that BE originates from the gastric cardia and that EAC arises from undifferentiated BE cells.	[57]
	RNA, DNA, MET	BE, dysplasia	2022	A multi-omics analysis of a longitudinally sampled Barrett’s esophagus patient cohort identifies the impact of structural variants, including mobile elements, and the timing of molecular events during progression, identifying the potential for cancer beyond the clinically discernible phenotype.	[55]
	scRNA, ST	LGIN, HGIN, ESCC	2023	A comprehensive scRNA sequencing and ST study of 79 multistage esophageal lesions from 29 patients with ESCC revealed a gradual and significant loss of ANXA1 expression in epithelial cells due to its transcription factor KLF4 suppression along the lesion progression and underscores ANxA1/FPR2 signaling as an important crosstalk mechanism between epithelium and fibroblasts in promoting ESCC.	[73]
	High-resolution ST	LGIN, HGIN, ESCC	2025	By generating a single-cell resolution spatial transcriptomic atlas of 127 fields from 43 patients, the study showed that a proliferative, dedifferentiated epithelial subpopulation drives ESCC progression by recruiting fibroblasts through JAG1–NOTCH1 signaling to form a protective CAF-epithelial niche at the tumor edge—an emergent microenvironmental hallmark that predicts disease advancement and patient outcomes.	[84]
Head and neck	scRNA	Leukoplakia, HNSCC	2023	Regulatory T cells in leukoplakia and HNSCC tissues were reported to express LAIR2, providing a favorable environment for tumor growth, and updating the pathobiological insights into cell–cell interactions during the stepwise progression of H NSCCs.	[183]
	scRNA, ST	Dysplasia, OSCC	2023	A single-cell transcriptome atlas and a spatial transcriptome map were built after obtaining data from pairwise human oral mucosal biopsies of 9 individuals consisting of very early-stage OSCC, adjacent precancerous lesions with moderate to severe dysplasia, as well as a matched normal region and an altered epithelial gene-expression profile was identified that favored OSCC initiation.	[69]
Intestinal	MET	Adenoma, CRC	2014	Genome-wide alterations in DNA methylation occur during early stages of progression of tubular adenomas to cancer, revealing heterogeneity in the pathogenesis of colorectal cancer, even at the adenoma step of the process.	[184]
	scRNA	Polyps, CRC	2021	A multi-omics atlas provided insights into malignant progression of colorectal polyps and their microenvironment, serving as a framework for precision surveillance and prevention of CRC.	[7]
	scRNA, scATAC	Polyps, CRC	2021	A continuum of epigenetic and transcriptional changes occurring in these stem-like cells as they progress from homeostasis to CRC was defined, further identifying regulatory markers for molecular staging of polyps.	[12]
	DNA, ATAC	Adenoma, CRC	2021	This study provided a map of (epi)genetic tumor heterogeneity, with fundamental implications for understanding colorectal cancer biology, using spatial multi-omics profiling of individual glands.	[39]
	ST, SP, DNA	Adenoma, CRC	2023	A transition to immune exclusion in CIN^+^ tumors as characterized by a novel gene expression signature composed of DDR1, TGFBI, PAK4, and DPEP1 is observed, demonstrating how these genes and their protein products are key regulators of extracellular matrix components, are associated with lower cytotoxic immune infiltration, and show prognostic value in external cohorts.	[83]
	scRNA	IBD	2024	Systematic integration of 25 scRNA sequencing datasets spanning the entire healthy gastrointestinal tract in development and in adulthood leads to a healthy reference atlas with approximately 1.1 million cells and 136 fine-grained cell states.	[20]
	Barcoded DNA, scRNA	GEMM mouse	2024	The data suggested that colorectal precancer is often founded by many different lineages and highlight their cooperative interactions in the earliest stages of cancer formation, providing insights into opportunities for earlier intervention in colorectal cancer.	[185]
Liver	scRNA	Cirrhosis	2019	The transcriptomes of more than 100,000 single human cells were profile, yielding molecular definitions for non-parenchymal cell types that are found in healthy and cirrhotic human liver, and unanticipated aspects of the cellular and molecular basis of human organ fibrosis are dissected at a single-cell level.	[63]
	DNA	Cirrhosis	2019	It was shown that cirrhotic liver has a higher mutational burden than normal liver, and structural variants, including chromothripsis, were prominent in cirrhosis.	[186]
	RNA	Cirrhosis, HCC	2019	An immune-related gene expression pattern in liver tissues of patients with early-stage HCC, called the ICF, was identified that associates with risk of HCC development in patients with cirrhosis.	[49]
	RNA, MS	NAFLD	2022	PLS-NAFLD was modified by bariatric surgery, lipophilic statin, and IDO1 inhibitor, suggesting that the signature can be used for drug discovery and as a surrogate end point in HCC chemoprevention clinical trials.	[187]
Lung	RNA	Metaplasia, dysplasia, CIS, LUSC	2019	It was proposed that carcinogenesis in the lung involves a dynamic co-evolution of pre-invasive bronchial cells and the immune response, and the need to develop immune biomarkers for early detection as well as immunotherapy-based chemopreventive approaches for individuals who are at high risk of developing lung cancer.	[47]
	scRNA, scATAC	GEMM mouse	2020	This study revealed a central principle underpinning intra-tumoral heterogeneity and motivates therapeutic targeting of the HPCS, which is associated with poor survival across human cancers and demonstrates chemoresistance in mice.	[66]
	scRNA	AAH, AIS, MIA, LUAD	2021	A group of cells closely resembling alveolar type 2 cells (AT2) that emerged during atypical adenomatous hyperplasia and whose transcriptional profile began to diverge from that of AT2 cells as LUAD progressed, taking on feature characteristic of stem-like cells, is detected.	[62]
	scRNA	GEMM mouse	2022	This study introduced an evolving lineage-tracing system with a single-cell RNA-seq readout into a mouse model of Kras;Trp53 (KP)-driven lung adenocarcinoma and tracked tumor evolution from single-transformed cells to metastatic tumors at unprecedented resolution.	[67]
	scRNA	LUAD, adjacent tissue	2024	The authors identified a novel KRT8^+^ alveolar intermediate cell population (KACs) with KRAS mutations and stem-like features that arises prior to LUAD formation, persists after carcinogen exposure, predicts poor survival, and may serve as an intermediate in AT2-to-tumor cell transformation and a target for KRAS-directed therapies.	[64]
	DNA, RNA, ST	AAH, AIS, MIA, LUAD	2025	Integrative analysis of multi-omics data revealed coordination between immune and nonimmune cells during early progression of precancerous lesions to lung adenocarcinomas and shed light on the molecular characteristics of clinically defined subtypes.	[56]
Pancreas	scRNA	IPMN	2018	The ability to perform high-resolution profiling of the transcriptomic changes that occur during multistep progression of cystic PDAC precursors to cancer was demonstrated and might be a useful substrate to identify targets for cancer interception.	[61]
	scRNA, scATAC	GEMM mouse	2023	The results uncovered a neoplasia-specific tissue-remodeling program that may be exploited for pancreatic cancer interception, and defining and quantifying epigenetic plasticity as the diversity in transcriptional phenotypes that is enabled or restricted by a given epigenetic accessibility landscape.	[70]
	scRNA	GEMM mouse	2023	It was demonstrated that MYC preferentially triggers transformation of the most immature MSI2^+^ pancreas cells into multi-lineage pre-cancer cells, which diverge to establish pancreatic cancer subtypes by activating distinct transcriptional programs and large-scale genomic changes.	[188]
	ST	PRAD, adjacent benign	2022	A systematic approach was used to study spatial genome integrity in situ and describe previously unidentified clonal relationships in benign and malignant tissue to suggest a model for how genomic instability arises in histologically benign tissue that may represent early events in cancer evolution.	[82]
	ST	PanIN	2024	It was demonstrated that cancer-associated fibroblasts (CAFs), including antigen-presenting CAFs, are located close to PanINs, and a transition from CAF-related inflammatory signaling to cellular proliferation during PanIN progression is observed.	[79]
Prostate	scRNA	GEMM mouse	2023	It was suggested that ETS overexpression alone, at sufficient dosage, can initiate prostate neoplasia.	[189]
Skin	DNA	Melanoma, nevus	2015	The succession of genetic alterations during melanoma progression was defined, showing distinct evolutionary trajectories for different melanoma subtypes, and an intermediate category of melanocytic neoplasia was identified, characterized by the presence of more than one pathogenic genetic alteration and distinctive histopathological features.	[190]
DNA, RNA	Melanoma, nevus	2018	Genomic and transcriptomic changes that accompany the evolution of melanoma from pre-malignant lesions were elucidated by sequencing DNA and RNA from primary melanomas and their adjacent precursors, as well as matched primary tumors and regional metastases.	[40]
SP, ST	Melanoma in situ, melanoma	2022	It was found that recurrent cellular neighborhoods involving tumor, immune, and stromal cells change significantly along a progression axis involving precursor states, melanoma in situ, and invasive tumor.	[191]
Stomach	DNA	LGD, HGD, GC	2010	Investigating copy number alterations (CNAs) of 20 gastric CISs and 20 adenomas found that the pattern of CNAs in HGA was quite different from that in LGA, suggesting that 8q gain is important for the malignant transformation of gastric adenoma.	[30]
	DNA, MET	GIM	2018	Several IMs exhibit hypermethylation at DNA methylation valleys; however, IMs generally lack intragenic hypomethylation signatures of advanced malignancy, and patients exhibiting normal-like epigenomic patterns were associated with regression.	[28]
	scRNA	Gastritis, GIM, GC	2019	A single-cell network underlying cellular and molecular characteristics of gastric epithelial cells across different lesions was constructed, and it was found that gland mucous cells tended to acquire an intestinal-like stem cell phenotype during metaplasia, and OR51E1 as a marker for unique endocrine cells in the early malignant lesion.	[59]
	scRNA	Organoid	2023	The model of occult preneoplasia by biallelic inactivation of TP53, a common early event in gastric cancer, in human gastric organoids implies predictability in the earliest stages of tumorigenesis and shows evolutionary constraints and barriers to malignant transformation.	[91]
	scRNA, ST	GIM, GC	2023	This study defines transcriptomic and spatial subtypes of intestinal metaplasia—one marked by an intestinal stem-cell-dominant compartment—and shows that clinical-genomic models, together with signatures of microbial dysbiosis, more accurately predict which IM lesions will progress to gastric cancer, highlighting new avenues for precision prevention.	[36]

### Bulk omics profiling

The initiation and progression of PMLs involve multi-layered molecular events, such as genomic, transcriptomic, metabolomic, and epigenomic alterations. Over the past 2 decades, bulk multi-omics profiling has become a widely adopted approach for characterizing these molecular changes. Among them, genomic sequencing has been the earliest and most widely used tool for investigating the driving mechanisms underlying premalignant evolution, either longitudinally [28,29] or cross-sectionally [30,31]. It has been frequently reported that many well-established driver genes, such as TP53 [32,33], APC, KRAS [34,35], and MYC [28], are also altered early at the PML stage and associated with later progression. As sequencing becomes more affordable and sensitive, researchers are now able to conduct large-scale, population-level genotyping, or clone-level evolutionary trajectory profiling of PMLs. For example, Huang et al. [36] performed high-depth targeted DNA-seq on 1,217 prospective GIM samples, systematically revealing the genomic heterogeneity of GIM. Chang et al. [37] conducted whole-genome sequencing (WGS) on 1,275 micro-biopsies of esophageal PMLs from 42 patients, uncovering the TP53-centered evolutionary patterns. In parallel, epigenomic alterations in PMLs also exhibit certain evolutionary patterns. At the methylation level, many PMLs show CpG focal hypermethylation at an earlier stage, whereas widespread hypomethylation, which may link to chromosomal instability, tends to accumulate during later stages of PMLs [28,38]. Notably, genome and epigenome often exhibit correlated and synergistic evolutionary paths, suggesting the presence of co-evolution [38,39]. In summary, bulk sequencing reveals that heritable alterations—including genomic-level SNVs, CNVs, and epigenomic-level DNA methylation and chromatin accessibility—together play pivotal roles in driving the progression from premalignancy to malignancy.

On the other hand, PML progression is not solely driven by individual molecular alterations, but also involves the aberrant activation of multiple cancer-associated signaling pathways or hallmarks. Transcriptomic, proteomic, and metabolomic profiling can capture these functional changes at the premalignant stages. For example, transcriptomics have uncovered that the MAPK signaling pathway is aberrantly activated early in nevi [40], whereas Wnt signaling in adenoma [41], AK [42], and BE [43]. Inflammation, as one of the major tumor-promoting hallmarks [2], often cooperates with driver mutations and contributes to tumorigenesis initiation, helping the acquisition of other cancer hallmarks [44,45], or even the acquisition of driver mutations themselves [46]. In later stages of PMLs, the initially activated immune and inflammatory responses generally shift toward an immunosuppression microenvironment, resulting in immune evasion and impaired clearance of mutated cells, as demonstrated by RNA-seq profiling in lung [47], colon [48], and liver [49]. Another substantial alteration in PMLs is metabolic reprogramming, including shifts in energy metabolism and the emergence of cancer-related metabolites [50]. For instance, aerobic metabolic pathways such as oxidative phosphorylation are transiently upregulated in low-grade LUSC but decrease in high-grade lesions [47], supporting the initiation of Warburg effect at a later stage. Likewise, metabolomic evidence showed the increase of glycolytic intermediates in cervix high-grade squamous intraepithelial lesion [51]. Notably, the microbiome has been increasingly recognized as playing a marked role in PML progression and tumorigenesis, as revealed by metagenomic sequencing [52]. For example, one of the earliest studies demonstrated specific tongue coating microbiota associated with the progression of gastritis [53], and fecal metagenomic analyses indicate that bile acids may stimulate certain microbiota, thereby promoting further carcinogenesis of colorectal adenoma [54]. Last but not least, recent PML-related multi-omics studies have provided greater details of the tumorigenesis process [9,55,56]. For example, Esplin et al. [9] collected 93 multi-regional colon samples from 6 familial adenomatous polyposis (FAP) patients with varying degrees of dysplasia and performed RNA-seq, proteomics, and metabolomics profiling. Multi-omics joint analysis inferred a latent axis correlated with dysplastic progression, comprising several co-varying molecules known to be associated with early tumorigenesis. The authors also found that the arachidonic acid pathway was up-regulated early in hyperplasia at both transcriptomic and metabolic levels, which was targeted by aspirin and other nonsteroidal anti-inflammatory drugs (NSAIDs), giving a potential explanation of the investigated preventative treatment mechanisms. In summary, bulk omics analyses of both intra- and inter-stage molecular heterogeneity in PMLs have provided critical insights into the mechanisms of PML progression.

Although bulk multi-omics profiling has greatly advanced our understanding of the biological mechanisms underlying tumor initiation from PMLs, the process of tumorigenesis is inherently complex and involves the coordinated participation of both premalignant cells and the microenvironment. In this regard, bulk approaches are limited by their tissue-level resolution and cannot resolve cellular heterogeneity. In particular, since tumorigenesis is fundamentally driven by a small subset of transformed cells, bulk sequencing overlooks critical cell-level information, thereby limiting our ability to gain deeper insights into the earliest events of malignant transformation.

### Single-cell omics profiling

Fortunately, advances in single-cell omics technologies over the past decade have provided unprecedented opportunities to dissect the cellular heterogeneity of PMLs and elucidate cell type-specific mechanisms that drive disease progression. Moreover, the ability of single-cell multi-omics approaches to profile large numbers of individual cells enables the precise pinpointing of rare cell populations within premalignant tissues that may serve as tumor-initiating cells (TICs), thereby allowing investigation of tumorigenesis at single-cell resolution. In addition, these technologies facilitate the study of intercellular crosstalks, providing critical insights into the complex cellular interactive networks that drive tumorigenesis.

With the recent maturation of single-cell technologies, an increasing number of studies have constructed single-cell atlases spanning normal, premalignant, and malignant stages across diverse organs, offering comprehensive insights into the cellular biology underlying PMLs. For instance, in gastrointestinal PMLs, metaplasia represents a common pathological feature, and scRNA-seq has been instrumental in elucidating its cellular origins. In the esophagus, Nowicki-Osuch et al. [57] revealed that BE arises from undifferentiated gastric cardia cells, driven by c-MYC and HNF4A, rather than from esophageal squamous cells. Similarly, Singh et al. [58] found that BE cells harbor a hybrid stomach and intestinal state at the chromatin level through scATAC-seq. Our prior research in the stomach identified a population of gland mucous cells with elevated expression of embryonic genes (e.g., ODAM and LEFTY1) as the probable origin of GIM, a result subsequently validated by independent studies [59,60]. Recently, Oliver et al. [20] systematically integrated 25 gastrointestinal single-cell datasets and discovered stomach-like metaplastic cells emerging from intestinal stem cells in IBD. Collectively, these findings underscore the link between metaplasia and dysregulated differentiation programs of adult stem cells. Single-cell atlases have also been established for other PMLs, such as IPMN [61], pulmonary AAH/AIS [62], and liver cirrhosis [63]. This single-cell profiling of PMLs provides critical insights into the cellular mechanisms underlying premalignant progression.

As previously noted, identifying rare TICs is a fundamental challenge in elucidating the mechanisms of tumorigenesis and constitutes a pivotal opportunity for early cancer intervention. Single-cell technologies offer unique advantages for detecting and characterizing TICs within PML samples. For instance, we and others have identified a population of metaplastic stem-like cells in PMLs of stomach that exhibit the highest transcriptomic similarity to early gastric cancer cells and increase in abundance along the progression cascade, suggesting a tumor-initiating role [36,59]. In the esophagus, Nowicki-Osuch et al. [57] demonstrated that esophageal adenocarcinoma (EAC) may arise from undifferentiated BE cells, even in the absence of histologically apparent BE tissue. Supporting the concept of transformation from PML stem-like cells to cancer cells, a continuum of stem-like populations spanning normal tissue, polyps, and CRC has been observed in both scATAC-seq and scRNA-seq space [12]. Alternatively, mature cells within PMLs can also serve as cells of origin for tumors. For example, Chen et al. [7] found that conventional adenomas progress to microsatellite stable tumors through WNT-driven stem cell expansion, whereas serrated polyps originate from terminally differentiated cells undergoing gastric metaplasia, ultimately giving rise to microsatellite instability-high tumors.

Given the rarity of TICs in cancer-free PML samples and the difficulty of longitudinal monitoring, adjacent non-tumor tissues with known malignant outcomes, as well as mouse and organoid models of tumorigenesis, can serve as accessible surrogates for investigating longitudinal progression. Recently, a representative work by Han et al. [64] applied single-cell and spatial transcriptomics on multi-region sampling of adjacent, intermediate, and distant LUAD biopsies, and identified a population of KRT8^+^ alveolar intermediate cells (KACs) characterized by reduced differentiation, enhanced plasticity. These cells exhibit elevated KRAS and CNV mutation burden, conferring tumor-initiating potential. They further validated the importance of KACs in tobacco-exposed and lineage-traced mice, and demonstrated that KAC-enriched organoid exhibited sensitivity to KRAS inhibition, inspiring the derivation of intervention targets. Since KRT8^+^ cells are enriched during tissue injury [65], this finding suggests that TICs may originate from highly plastic cells generated during injury repair processes, such as those following tobacco exposure. Likewise, the Kras/p53 (KP) mouse model of LUAD also possesses a highly plastic cell state, which arises from a mixed AT1/AT2 phenotype, and exhibits high tumorigenic potential [66,67]. In summary, single-cell studies have facilitated the identification of TICs originating from stem cells, mature cells, or highly plastic cell states, which may be further exploited as early diagnostic markers or therapeutic targets at the cellular level.

In addition to TICs, microenvironmental cells and their interactions with transformed epithelial cells play pivotal roles in the process of tumorigenesis. As previously discussed, the premalignant stage is marked by a transition from immune activation to immunosuppression; single-cell analyses have further contextualized this shift within specific immune cell types. For example, in low-grade IPMN, pro-inflammatory cell types such as cytotoxic T cells and activated T helper cells are abundant, but their prevalence diminishes in later stages, accompanied by increased infiltration of myeloid-derived suppressor cells [61]. Moreover, regulatory T cells—recognized as a primary driver of T cell exhaustion—emerge during the premalignant stage and contribute to the accumulation of exhausted T cells [12]. Notably, intercellular interactions are critical for remodeling the microenvironment in ways that facilitate tumorigenesis [68]. For instance, a previous study demonstrated that INHBA^+^ monocytes are enriched in dysplastic leukoplakia and have extensive interactions with exhausted CD8^+^ T cells, and targeting these 2 cell populations in mouse models resulted in significantly less severe lesions [69]. Furthermore, Burdziak et al. [70] identified an IL-33-mediated inflammatory feedback loop between highly plastic epithelial cells and immune cells, and functional experiments demonstrated that IL-33 signaling drives the exit from highly plastic states and promotes progression toward tumorigenesis, highlighting the critical role of epithelium–microenvironment crosstalk in tumor initiation.

On the other hand, fibroblasts in the microenvironment can be reprogrammed into cancer-associated fibroblasts (CAFs), particularly myofibroblastic CAFs (myCAFs), during the later stages of PMLs [71,72]. These myCAFs orchestrate extracellular matrix remodeling, release sequestered cytokines, and facilitate cellular migration and invasion; moreover, they possess immunomodulatory capacity that promotes immune evasion [68]. For instance, Chen et al. [73] demonstrated that in esophageal HGIN, epithelial cells exhibit diminished ANXA1 secretion, disrupting homeostatic interaction with FPR2 receptors on fibroblasts and driving their transition to myCAFs. Other microenvironmental cell types, including antigen-presenting neutrophils [74] and angiogenesis-promoting endothelial cells [75], also contribute to the tumor microenvironment, although their tumor-promoting or suppressive roles in PMLs remain incompletely understood. Given that microenvironmental cells exhibit higher cross-organ similarity than epithelial cells, future integration of single-cell atlases across organs is expected to yield a more comprehensive understanding of the dynamic evolution of microenvironmental populations in PMLs [13,14]. Collectively, from delineating the cellular-resolution landscape of PML evolution, to identifying TICs, and elucidating co-evolution with microenvironmental cells, single-cell omics have fundamentally transformed the paradigm of PML research.

### Spatial omics profiling

In parallel with the widespread adoption of single-cell omics, spatially resolved omics technologies have rapidly emerged in recent years [76]. Single-cell omics approaches, which require tissue dissociation, inevitably disrupt the spatial context and cell–cell proximity information that are essential for understanding tumorigenesis. In contrast, spatial omics preserves the native histological architecture during sequencing, thereby enabling spatially resolved observation of cellular and clonal evolution in PMLs. Furthermore, as many cell–cell interactions occur through direct contact or paracrine signaling, the retention of spatial proximity information is instrumental in dissecting the complex interplay among diverse cell types within tissues [77]. Moreover, since histopathological annotation of hematoxylin and eosin (H&E) images in many PMLs is performed at the level of individual glands, spatial omics enables the investigation of relationships between glandular phenotypic alterations and underlying molecular changes, thereby providing valuable insights for precise clinical diagnosis and risk stratification of PMLs. Consequently, spatial omics enables the tracing of the spatial evolution of putative TICs and the investigation of their associated local niches, thus offering a more comprehensive understanding of PML progression and tumor initiation [15].

Among the spatial omics technologies, sequencing-based spatial transcriptomics (ST) methods, such as Visium, Slide-seq, and Digital Spatial Profiling (DSP) are particularly widely used in PML research, offering varying degrees of coverage and resolution [78]. For example, Visium-based ST and additional imaging analysis of PanIN revealed that CAF subtypes adjacent to PanIN lesions closely resemble those found in invasive PDAC, and as PanIN progresses, CAF-associated inflammatory signals shift toward proliferative signaling [79]. Slide-seq of healthy and dysplastic colon identified 3 distinct multicellular community regions: inflammatory epithelial zones, epithelial stem cell-like regions, and dysplastic cell areas, each enriched with specific functional microenvironmental cell subtypes [80]. On the other hand, DSP requires selecting regions of interest beforehand and is suitable for studying region-level histopathologic heterogeneities. For instance, DSP of IPMN regions with different subtypes and dysplasia severity uncovered the lineage relationships and molecular markers of high-risk IPMN [81].

Notably, during tumor evolution, adjacent cellular clones often share a recent common ancestor. Therefore, the retention of spatial information in ST is particularly advantageous for reconstructing the evolutionary trajectories of TICs in both malignant and adjacent non-malignant tissues. For instance, Erickson et al. [82] utilized transcriptome-based inference of CNV and clonal relationships to delineate distinct spatial clonal architectures within tumors and adjacent benign tissues across multiple organs, highlighting the widespread presence of genomic instability in PML tissues. Moreover, Heiser et al. selected 31 CRC specimens with concurrent pre-malignant and malignant regions, and integrated CNV inferred from ST data with micro-biopsy WES to reconstruct phylogenetic relationships. They demonstrated that adenoma-to-carcinoma progression in the colon could be classified into 2 evolutionary trajectories: chromosomal instability (CIN^+^) and hypermutated pathways [83]. The CIN^+^ trajectory is characterized by branched and neutral evolution, whereas the hypermutated pathway exhibits linear evolution dominated by a single clone. By further incorporating multiplexed protein imaging to assess microenvironmental cell dynamics along these evolutionary trajectories, they found that CIN^+^ tumors tended to exhibit immune-cold phenotypes, mediated by multiple immune exclusion signals. This study exemplifies the power of spatial omics in elucidating the co-evolution of epithelial and microenvironmental compartments throughout tumorigenesis.

In contrast, imaging-based low-throughput spatial profiling methods, although typically limited to detecting a few dozens of genes, are capable of achieving single-cell or even subcellular spatial resolution. These approaches are especially advantageous when the main goal is to elucidate the spatial organization of cell types or to interrogate a targeted set of genes, rather than to obtain a comprehensive molecular landscape. For example, Risom et al. [8] utilized multiplexed ion beam imaging by time of flight (MIBI-TOF) with a 37-antibody panel to analyze 79 clinically annotated DCIS surgical specimens, and constructed a digital TME atlas encompassing 433 spatially resolved cellular features. Their analysis revealed that TME undergoes coordinated transitions among 4 distinct states during disease progression; notably, DCIS patients without recurrence exhibited greater disruption of the myoepithelial layer, suggesting a potentially protective role for myoepithelial cells in preventing recurrence. Recent technological advances have enabled in situ detection of hundreds to thousands of genes at single-cell resolution. For instance, Chang et al. [84] employed the Xenium In Situ and TF-seqFISH platform to profile up to 1,471 genes to investigate the progression from normal tissue to ESCC. They discovered that proliferative epithelial subpopulations drive ESCC progression by acquiring dedifferentiated and invasive properties. At the HGIN stage, these cells recruit normal fibroblasts through JAG1-NOTCH1 signaling, inducing their conversion to CAFs and establishing CAF–Epi niches at the tumor margin that protect tumor cells against immune surveillance. Thus, high-resolution spatial profiling enables the delineation of tumorigenesis with unprecedented detail, uncovering insights that were previously unattainable. In summary, advances in spatial omics technologies have substantially deepened our understanding of cellular and clonal evolution in PMLs, and their complex interactions with the surrounding microenvironment.

### Challenge in omics profiling of PMLs

As discussed above, the rapid advancement of multi-omics technologies has substantially accelerated research on PMLs across diverse organs. However, several key challenges continue to constrain the systematic omics characterization of PMLs and hamper downstream clinical translation. First, at the level of clinical specimens, given the central role of TICs in tumorigenesis, their rarity poses a major obstacle. On one hand, early premalignant foci are small, with limited quantities of obtainable tissues in biopsy, and during single-cell sequencing, the tissue used for pathologic diagnosis may not fully coincide with that used for omics assays. This mismatch can make putative TICs susceptible to omission due to sampling bias. On the other hand, longitudinal cohorts with outcome annotation (progression vs. non-progression) require years of accrual and pathological adjudication, which complicates cohort construction difficult and hinders robust causal inference between candidate TICs and tumor initiation. In this regard, several representative studies discussed above employed systematic multi-site sampling of tumor-adjacent tissues at proximal and distal locations as an alternative, obtaining PML tissues closely linked to tumorigenesis [64,83]. Moreover, recent formalin-fixed, paraffin-embedded (FFPE)-compatible single-cell and spatial transcriptomics technologies [85,86] enable retrospective identification of high-risk patients from archived specimens and delineation of high-risk regions guided by histopathologic annotation. Finally, multi-center consortia such as HTAN provide an enabling framework for building high-quality PML cohorts [14].

Second, at the technical level, the complex workflow—from PML sampling to sequencing and data processing—undermines the reproducibility of sequencing results, and the lack of standardized procedures further exacerbates batch effects, hindering the integration and reanalysis of tumorigenesis-related signals across studies. Furthermore, the high cost of single-cell and spatial sequencing inevitably creates a trade-off between cost, sample size, and technical replication, thereby reducing statistical power in downstream analyses. To address these issues, researchers have proposed several solutions. For instance, HTAN has established a centralized Data Coordinating Center to harmonize tumor-related multi-omics data, thereby enhancing reproducibility and data reuse across centers [87]. In addition, several data standards have been developed to align datasets with the principles of Findability, Accessibility, Interoperability, and Reusability (FAIR) [88,89]. Similarly, the HCA gastrointestinal atlas [20] introduced scAutoQC for standardized quality control of single-cell data, while in spatial transcriptomics, SpatialData [90] provides a unified data structure, lowering the barrier for cross-modal alignment and reanalysis. Collectively, these efforts establish feasible standardization paradigms for PML multi-omics research and hold promise for alleviating bottlenecks in reproducibility and scalability.

Finally, at the biological mechanism level, although multi-omics has increasingly identified key molecules and cell populations in PMLs, a substantial gap persists between correlative observation and mechanistic causation. As noted above, the lack of high-quality sequential cohorts of premalignant progression results in weak evidence for causal chains underlying tumor initiation. In this regard, advances in gene-editing technologies in recent years have made preclinical models, including mice and organoids, have become powerful alternatives for studying tumor initiation [91–93]. These models not only enable longitudinal sampling at multiple time points and continuous monitoring through imaging, but also facilitate lineage tracing and large-scale CRISPR screening [11,92]. However, they also face limitations such as high cost, long latency of malignant transformation, interspecies anatomical differences (e.g., the presence of a forestomach in mice that is absent in humans), and discrepancies in immune and stromal microenvironments, all of which warrant careful consideration. In summary, multi-omics studies of PMLs continue to face major challenges—including limited access to high-quality clinical specimens, insufficient standardization of sampling and data processing, and difficulty in mechanistic dissection—all of which warrant systematic resolution in future research.

## Computational Approaches for PML Multi-Omics

**Fig. 2.**
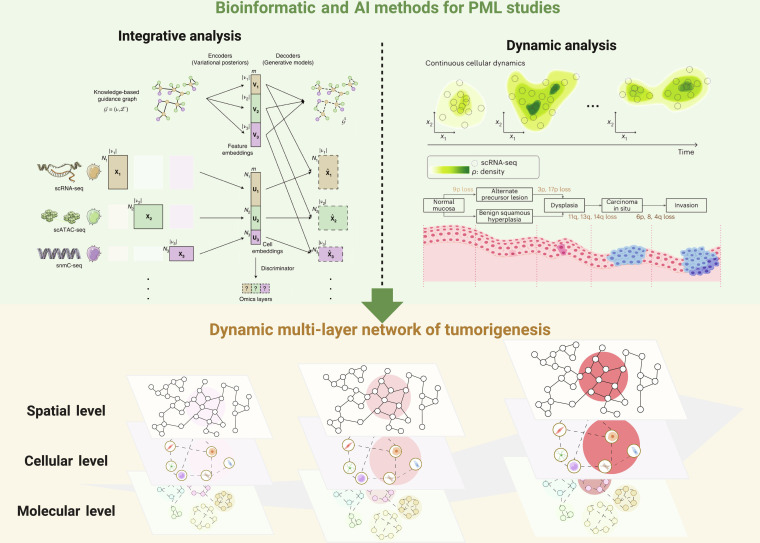
Computational methods for dissecting PML heterogeneity. The integrative analysis (upper left) can integrate information from multi-omics and learns complex interactions among them, while the dynamic analysis (upper right) models the gradual transformation during PML progression to tumors, including transcriptional modeling of continuous cellular dynamics and genetic modeling of discrete molecular alterations. Together, a dynamic, multi-layer network depiction of tumorigenesis —spanning the spatial (phenotypic/morphological), cellular, and molecular levels—can further illuminate its key mechanisms. Elements in the “Integrative analysis”, “Dynamic analysis” (top and bottom), and “Dynamic multi-layer network of tumorigenesis” panels are adapted from Cao and Gao [102], Sha et al. [116], Leshchiner et al. [119], and Zhang et al. [159], respectively. Figure created with Biorender.com.

The progression from PMLs to malignancy represents an intrinsically complex, multi-layered longitudinal process involving phenotypic, cellular, and molecular alterations. Advanced computational methodologies are indispensable for the effectively extracting tumorigenesis-associated signals from multi-omics data. From one perspective, diverse omics platforms capture complementary facets of tumorigenesis across varying resolutions and modalities, thereby necessitating robust integrative analyses. Conversely, omics investigations generally yield only discrete snapshots along the continuum from premalignancy to malignancy, necessitating the computational inference of continuous cellular and molecular dynamics from limited temporal observations. Accordingly, this section provides an overview of computational methodologies relevant to tumorigenesis research from 2 complementary perspectives: multi-omics integration and dynamic trajectory analysis (Fig. 2). Table 3 systematically compares these computational approaches, including representative tools, underlying principles, advantages and limitations, and suitable PML research scenarios.

**Table 3.** Summary of computational methods

Method category	Representative methods	Principles	Advantages	Limitations	PML research scenarios
**Deconvolution**					
Bulk deconvolution	CIBERSORT[94], BayesPrism [95]	Estimates cell-type proportions and/or gene expression profiles from bulk RNA-seq data using single-cell reference signatures.	Well-established and computationally efficient for analyzing large cohorts; enables inference of cellular heterogeneity from conventional bulk RNA-seq datasets.	Strongly reliant on the quality, completeness, and representativeness of reference atlases and marker gene selection; performs poorly with closely related or novel cell states.	Profiling shifts in immune or stromal infiltration within archival bulk RNA-seq datasets from longitudinal PML cohorts; identifying cell types correlated with progression.
ST deconvolution	RCTD[96]	Decomposes ST spots into constituent cell types by leveraging single-cell reference profiles and probabilistic modeling.	Enhances cellular resolution in ST datasets; elucidates the spatial organization of cell populations within tissues.	Shares reference dependency issues with bulk deconvolution; challenged by highly overlapping cell types, low-expression genes, and inherent limitations in spot size and capture efficiency.	Mapping the precise spatial localization of immune cells (e.g., regulatory T cells) or epithelial subclones within premalignant niches.
**Single-cell mapping**					
Bulk to single cell	Scissor[98]	Correlates single-cell profiles with bulk phenotypic data to pinpoint subpopulations linked to clinical outcomes via regression-based modeling.	Bridges scRNA-seq atlases with bulk phenotypes without requiring paired samples.	Necessitates robust bulk cohorts with well-defined phenotypes; detects correlations rather than causal drivers.	Identifying epithelial or immune subpopulations associated with progression or regression in scRNA-seq atlases of PMLs, utilizing bulk data from patients with documented outcomes.
Spatial to single cell	CellTrek[99], CytoSPACE[100]	Assigns spatial coordinates to single cells based on transcriptomic similarity to ST or histological references, employing co-embedding or optimization techniques.	Achieves near-single-cell spatial resolution; facilitates spatial analyses of comprehensive scRNA-seq datasets.	Computationally demanding for large-scale data; mapping precision may vary due to technical artifacts.	Reconstructing detailed spatial topologies of premalignant subclones and their microenvironments to investigate early spatial interactions and niche dynamics.
**Multi-omics integration**					
Probabilistic and matrix factorization	MOFA[101]	Factorizes multi-omics data into latent components capturing shared and modality-specific variations via probabilistic or matrix decomposition approaches.	Yields interpretable latent factors; accommodates missing data; proven effective for bulk multi-omics analyses.	Scales inadequately to expansive single-cell datasets; challenged by high sparsity levels.	Identifying coordinated molecular signatures across omics layers that characterize high-risk PML states in bulk tissue specimens.
Deep learning (VAE-based)	GLUE[102], MIDAS[103]	Uses variational autoencoders to learn a joint latent representation that integrates multiple modalities.	Handles noise, sparsity, and large cell numbers well; flexible for various modalities.	“Black-box” nature can reduce interpretability; training can be complex and require substantial data.	Large-scale integration of scRNA-seq and scATAC-seq to infer key transcription factors driving PML initiation.
Deep learning (transformer/LLM-based)	Geneformer[105], scGPT[106]	Leverages transformer architectures pretrained on vast scRNA-seq corpora to acquire foundational biological embeddings for multi-omics tasks.	Facilitates potent transfer learning; excels in downstream applications with limited fine-tuning; captures intricate contextual dependencies.	Incurs substantial computational overhead for pretraining; currently predominantly transcriptomics-oriented.	Developing foundational models of premalignancy through fine-tuning on PML atlases for applications such as risk stratification or therapeutic response prediction.
Deep learning for spatial integration	Tangram[192], STAligner[193]	Employs deep learning architectures to align single-cell and spatial data into a unified latent space for integration and imputation.	Robustly addresses batch effects and data complexity; often outperforms traditional methods in mapping and integration accuracy.	Models are intricate and resource-intensive; interpretability may be constrained.	Constructing a cohesive, high-resolution spatial atlas of PMLs by integrating multi-patient samples to uncover conserved spatial motifs.
Gene regulatory network inference	SCENIC[194], LINGER[104]	Reconstructs context-specific GRNs or interaction networks by integrating omics data with prior knowledge bases (e.g., transcription factor motifs).	Generates mechanistic insights by pinpointing key drivers and regulatory modules.	Heavily dependent on the comprehensiveness of prior knowledge resources; typically yields static network representations.	Delineating core transcriptional regulators and rewired network components that mediate transitions from normal to precancerous and cancerous states.
**Multimodal integration (image + omics)**					
Image-based omics prediction	DeepPT[195]	Establishes paired embeddings between hematoxylin and eosin (H&E) image patches and omics profiles using deep neural networks.	Enables prediction of molecular features from routine H&E images and holds substantial potential for clinical translation.	Gene-specific prediction accuracy may be moderate; requires large, well-paired training datasets.	Forecasting spatial molecular patterns or immune infiltration from standard H&E slides of PMLs to facilitate cost-effective biomarker identification.
Image-based cell typing	HistoCell[111]	Trains models to classify cell states from H&E images using ST-derived cell type proportions as weak supervisory signals.	Permits identification of cell types or states from histology without precise pixel-level annotations.	Efficacy hinges on ST reference quality; restricted to cell types represented in the reference.	Enabling digital pathology-based screening to detect and quantify high-risk cell populations (e.g., TICs, exhausted T cells) in extensive H&E image archives from PML biopsies.
**Dynamic process inference**					
RNA velocity/trajectory inference	scVelo[114]	Infers transcriptional dynamics and cell state transitions from unspliced/spliced mRNA ratios, modeling directionality and velocity.	Derived directly from empirical data without a priori assumptions; elucidates potential lineage hierarchies.	Vulnerable to assumptions regarding RNA kinetics and data quality; interpretations may be equivocal in multifaceted datasets.	Deducing lineage relationships and directional differentiation among normal, premalignant, and malignant epithelial states in scRNA-seq datasets.
Optimal transport (OT)	TIGON[116]	Simulates probabilistic mass transport (cells) across states or locations by optimizing a cost function, incorporating spatial and temporal constraints.	Provides a versatile, mathematically grounded framework for trajectory reconstruction.	Computationally burdensome; demands meticulous cost metric specification.	Reconstructing immune cell migration paths into PML niches or spatial expansion trajectories of evolving epithelial clones.
Phylogenetic inference	PhylogicNDT[119], CalicoST[121]	Constructs evolutionary phylogenies of cell lineages using somatic mutations (e.g., single-nucleotide variants or copy-number variations) as phylogenetic markers.	Offers a direct chronicle of clonal evolution anchored in immutable genetic changes.	Requires high-fidelity DNA sequencing (e.g., deep coverage); may overlook convergent evolutionary phenomena.	Tracing clonal origins and evolutionary trajectories in PMLs, elucidating the sequence of driver mutations, and spatially mapping subclones in histological contexts.

### Integrative analysis of multi-omics PML data

From a technological perspective, different omics platforms exhibit unique trade-offs regarding cost, sequencing throughput, and resolution. Consequently, the integration of complementary omics approaches and joint analytical strategies consititutes an effective solution. For instance, single-cell data are frequently leveraged to guide the deconvolution of bulk datasets, enabling the estimation of cell type-specific contributions [94,95]. Similarly, since each spot in ST data generally comprises tens to hundreds of cells, deconvolution approaches can aid in recovering single-cell-level resolution [96,97]. Another promising avenue involves using bulk or spatial data to inform the analysis of single-cell datasets. For example, the Scissor [98] method employs phenotype-associated bulk expression data to train regression models on the correlation matrix between bulk samples and single cells, thereby identifying single-cell subpopulations linked to specific biological or clinical features. By leveraging bulk datasets with definitive progression or regression outcomes, key cell populations associated with PML progression can be identified within single-cell atlases that lack explicit outcome annotations. CellTrek [99] and CytoSPACE [100] instead map single cells back to spatial coordinates—rather than deconvoluting spatial data—by employing co-embedding strategies and convex optimization, respectively. The authors of CellTrek further applied their method to DCIS samples, revealing that distinct DCIS subclones were mapped to different ductal regions, and that Treg and exhausted T cells were localized around tumor cells. These findings suggest that such approaches can reconstruct the spatial topology of multiple premalignant subclones and their associated microenvironments, thereby enabling the investigation of coordinated spatial evolution between epithelial and microenvironmental cells.

Moreover, each omics layer—such as the genome, transcriptome, or proteome—captures only a specific aspect of PML progression. The biological essence of disease progression is determined not by the simple summation of multiple layers, but by the intricate interactions among them. Therefore, integrative multi-omics analysis can uncover patterns that are inaccessible to single-omics approaches. Classical statistical approaches, such as probabilistic modeling and matrix factorization, have played a pivotal role in bulk multi-omics integration [101]. For example, Esplin et al. [9] applied the MOFA framework to integrate 4 omics layers from FAP patients, identifying a latent axis correlated with dysplastic progression, as previously mentioned. However, traditional methods struggle to accommodate the large cell numbers, high data sparsity, and pronounced batch effects in single-cell datasets, and are limited in modeling complex inter-omics interactions. In this context, deep learning approaches have recently demonstrated superior accuracy and scalability for single-cell multi-omics integration, enabling the extraction of key patterns from complex and heterogeneous data [102,103]. For example, MIDAS integrates variational autoencoder (VAE), self-supervised learning, and information-theoretic approaches to enable large-scale mosaic integration of single-cell multi-modal data [103], thereby facilitating the construction of comprehensive precancer atlases. Moreover, integrative analysis of RNA and ATAC data enables the effective inference of key upstream transcription factors (TFs) and their associated gene regulatory networks (GRNs) relevant to PML progression [12]. For example, LINGER integrates paired single-cell RNA and ATAC data, incorporates large-scale external bulk datasets and TF binding motif knowledge, achieving state-of-the-art accuracy in GRN inference [104]. Last but not least, foundation models that leverage transformer architectures and massive single-cell datasets enable broad data comprehension and strong adaptability to downstream tasks through large-scale pretraining paradigms [105,106]. Recently, Fu et al. [107] pretrained on extensive scATAC-seq data and fine-tuned on paired single-cell RNA and ATAC datasets to develop an interpretable foundation model that uncovers regulatory grammars, infers gene regulation and TF interactions, and even predicts gene expression in a zero-shot setting, which may facilitate the systematic identification of gene regulatory events underlying tumorigenesis. In summary, deep learning-based multimodal approaches utilizing VAE and transformer architectures demonstrate strong capabilities for integrative analysis of single-cell multi-omics data, and are positioned to introduce novel paradigms for multi-omics research in PMLs. Nevertheless, the black-box nature of neural network-based approaches, their high computational demands, and the potential risk of overfitting remain non-negligible. When developing or selecting appropriate tools, considerations should extend beyond performance to include accessibility and the interpretability of results.

Another promising direction involves cross-modal association learning between H&E-stained pathological images and omics data using deep learning approaches, which can elucidate the relationship between tissue morphology and molecular expression; or predict spatial molecular profiles and cellular distributions from histopathological images, offering substantial clinical potential. For example, transformer-based models have achieved an AUC of up to 0.99 in predicting microsatellite instability (MSI) status in CRC directly from H&E images [108]. Moreover, SCHAF employs adversarial autoencoders to learn paired representations between single-cell transcriptomes and H&E-stained images, enabling the direct generation of spatially resolved single-cell omics datasets from histopathological images [109]. However, the average correlation between gene expression predicted from H&E images and experimentally observed values remains modest (approximately 0.25) [110], which limits the practical utility of such predictions in downstream analyses. Recently, our laboratory developed HistoCell, which utilizes cell-type proportions inferred from ST data as weak supervision signals to train deep neural networks that infer cell types or states from H&E image features, achieving relatively high accuracy [111]. Furthermore, additional pretraining on ST data and H&E images from patients with gastric early-malignant lesions and concurrent LGD foci demonstrated that HistoCell can effectively infer potentially high-risk cell populations in LGD, highlighting its potential for clinical cancer risk screening. We anticipate that with enhanced input data quality, advanced model architectures, and systematic evaluation frameworks, deep learning-based multimodal integrative approaches will continue to yield novel biological discoveries and clinical insights in PML research.

### Dynamic modeling of tumorigenesis

While integrative analysis addresses heterogeneity across different omics layers, dynamic modeling focuses on temporal heterogeneity and the evolving nature of tumorigenesis. Given that tumorigenesis is an inherently complex and sequential process, dynamic modeling provides a more precise framework for elucidating the temporal evolution of tumor development [112]. Depending on the data types leveraged for inferring dynamics, dynamic modeling methods can be broadly categorized into transcriptome-based trajectory inference and genome-based clonal evolution reconstruction.

Omics data typically provide only single-time-point observations of individual cells, thereby precluding continuous temporal monitoring. Consequently, trajectory analysis frequently leverages spatially preserved patterns of cellular differentiation within individual glands or progressive evolution across multiple glands. For example, Becker et al. investigated the adenoma–carcinoma sequence in the colon and observed that, as lesions progress, cells exhibit a tendency to shift toward stem cell-like states at the initiation of the trajectory. They further discovered that the stem cell population itself undergoes a continuum of transformation from normal-like stem cells to CRC-associated phenotypes, characterized by elevated GPX2 expression [12]. Trajectory inference methods based on cell-to-cell similarity [113] can concurrently model both differentiation and evolutionary dynamics. However, the resultant trajectories may conflate these distinct processes, potentially leading to interpretational ambiguity. In contrast, RNA velocity-based approaches [114] can distinguish differentiation-specific RNA splicing dynamics, thereby enabling the separation of differentiation processes from other evolutionary mechanisms. Recently, optimal transport (OT)-based methods have gained prominence owing to their robust modeling capacity and computationally efficient neural network optimization. These approaches provide distinctive advantages for trajectory reconstruction [115]. For instance, TIGON [116] employs dynamic unbalanced OT modeling and neural ordinary differential equation solvers to recover cell velocities accurately and reconstruct trajectories and growth patterns, and infer temporal GRNs and cell–cell communication. As additional PML datasets become available, these advanced methodologies are anticipated to facilitate the identification of consensus cellular trajectory patterns underlying tumorigenesis.

Relying exclusively on transcriptomic data frequently presents challenges in establishing long-term associations, particularly due to the limited observation of intermediate states between PMLs and cancer. Consequently, inferring evolutionary trajectories or performing lineage tracing based on genomic signals can more precisely establish connections between tumors and their premalignant origins. For example, multi-region sampling of tumors and adjacent premalignant tissues could enable the reconstruction of tumorigenesis trajectories using classical phylogenetic tree algorithms [117]. Furthermore, given that higher clonal prevalence and multiplicity serve as indicators of earlier formation events, the temporal sequence of genetic alterations could also be inferred exclusively from the mutational profiles of tumors themselves [118]. For instance, Leshchiner et al. [119] developed PhylogicNDT and demonstrated its ability to estimate the relative timing of early driver mutations and HPV integration events in head and neck squamous cell carcinoma initiation. This underscores the potential of such methodologies to inform early detection or intervention strategies in tumors where premalignant tissues remain inaccessible. On the other hand, spatially resolved sequencing preserves clonal relationships in spatial dimensions, thereby facilitating the identification of clonally related regions that are also temporally adjacent in evolutionary progression. Consequently, clonal inference based on spatial omics methodologies offers additional advantages. As previously mentioned, Erickson et al. [82] utilized SpatialInferCNV to elucidate clonal evolutionary relationships between tumors and adjacent benign tissues. Lomakin et al. [120] proposed a spatial genetic clone mapping workflow called BaSISS, which integrates whole-genome sequencing, base-specific in situ sequencing, and ST data with specialized algorithms to infer ancestral relationships. Applying BaSISS to the DCIS–breast cancer transition, the authors revealed distinct transcriptional, histological, and microenvironmental patterns across subclone territories. Recently, Ma et al. [121] developed CalicoST, which directly infers allele-specific copy number variations from raw sequencing read alignments, integrating spatial coordinates to reconstruct evolutionary relationships, thereby substantially enhancing the capability to infer clonal dynamics from ST data. Finally, in addition to copy number variations, mitochondrial DNA (mtDNA) demonstrates particular suitability for evolutionary inference owing to its accelerated mutation rate. For instance, Gier et al. [122] leveraged mtDNA derived from single-cell RNA sequencing data to identify clonal cell states that bridge BE and EAC. Collectively, transcriptome-based trajectory inference facilitates continuous modeling of cellular dynamics throughout tumorigenesis, while genomic information preserved through spatial adjacency enables accurate reconstruction of PML-to-tumor clonal evolution.

## Clinical Translation of Omics Studies on PMLs

### Biomarkers for PML risk stratification

**Fig. 3.**
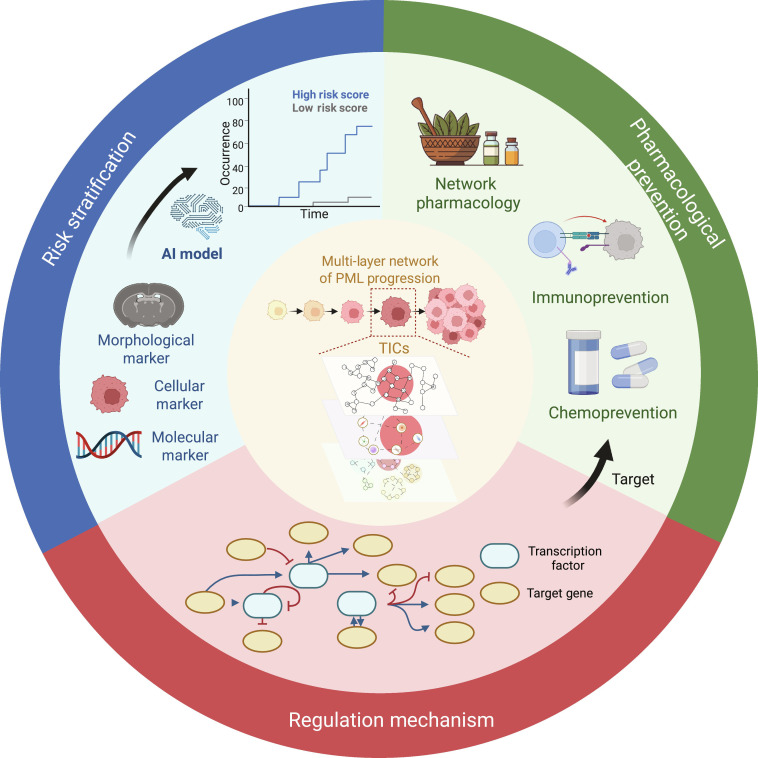
Clinical potential of premalignant studies. The central circle depicts the gradual transformation from normal cells to tumors, with a highlight of the multi-layer biological network underlying tumorigenesis mediated by TICs. The upper-left sector depicts a vision that integrates multiple sources of information using an AI model to accurately stratify the progression risk of premalignant patients. The lower sector is a schematic diagram of the regulatory mechanisms for tumorigenesis, in which certain TFs abnormally regulate downstream genes, providing insights for potential intervention targets. The upper-right sector lists 3 potential approaches for the pharmacological prevention of cancer: chemoprevention, immunoprevention, and network pharmacology approach. Created with BioRender.com. PML, premalignant lesion; TICs, tumor-initiating cells; TF, transcription factor.

The monitoring of PML progression has long been an unsolved challenge in clinical practice, as conventional modalities such as medical imaging and histopathological assessment continue to serve as the primary approaches, yet are often limited by suboptimal accuracy and high invasiveness for many organs. Canonical tumor markers, including CEA and CA19-9, similarly exhibit limited specificity and sensitivity for the detection of incipient tumors [123]. Recent advances in omics technologies have substantially contributed to the discovery of robust molecular markers for progression risk stratification among premalignant patients (Fig. 3). In the context of genomics, specific mutations or chromosomal aberrations within a PML, such as TP53 mutations or loss of heterozygosity (LOH) in BE [124], and 8q gain or telomere shortening in GIM [28], may serve as indicators of elevated progression risk. Beyond individual gene alterations, mutational signatures may also serve as biomarkers. For instance, scDNA-seq analyses of BE have identified the SBS17 mutational signature as being present in chromosomally unstable BE cells but absent in stable ones [125]. Compared to genomic markers that are generally stable but may be relatively infrequent in certain premalignant contexts, gene expression and epigenetic profiles provide a more dynamic reflection of disease progression. For example, Strand et al. [126] developed a classifier utilizing 812 differentially expressed genes to predict the risk of DCIS recurrence or invasive progression, achieving a hazard ratio of 7.3 in the validation cohort. Yang et al. [127] established a methylation-based mitotic-like clock that quantified the methylation level of 385 promoter CpG sites and showed that lung CIS patients who progressed exhibited an accelerated clock. Collectively, multi-omics approaches are facilitating the identification of biomarkers indicative of high-risk PMLs; nevertheless, considerable work remains to achieve comprehensive clinical validation and practical implementation.

Furthermore, omics technologies are enabling less invasive detection and monitoring of PMLs. For example, genome-wide methylation profiling identified CCNA1 and VIM as biomarkers for BE and EAC, which, when combined with swallowable-balloon sampling, have enabled practical, office-based screening without the need for endoscopy [128]. Apart from tissue biopsy, non-invasive liquid biopsies, such as cfDNA, exosomes, and metabolites in blood, have demonstrated considerable potential in tumor detection [129]; however, their application in PMLs remains limited due to lower tumor-derived material shedding and greater confounding by host factors compared to established tumors. One study integrated KRAS mutations with NDRG4 and BMP3 methylation markers in a stool DNA assay for screening of advanced adenoma and CRC, demonstrating significantly improved accuracy over fecal immunochemical testing, and received Food and Drug Administration approval and guideline endorsement [130]. Additionally, plasma metabolomic profiling has identified potential biomarkers for stratifying high-risk PML patients [131], which require further validation in multicenter and prospective studies. Of note, recent advances in ultrasensitive detection methodologies, coupled with machine learning-based signal enrichment, have enabled the detection of ctDNA in colorectal adenomas, thereby providing a template for future investigations [132].

Owing to rapid advancements in sequencing technologies, our understanding of biomarkers has evolved from the relatively coarse bulk and individual levels to single-cell and spatial resolution. Single-cell sequencing enables the delineation of specific cell types or states that emerge and transform during the progression from PMLs to cancer, while spatially resolved omics situates these cells within a morphologically and spatially contextualized framework. Such cell types or states can subsequently be leveraged as cellular biomarkers. For example, Risom et al. [8] extracted 433 features from MIBI-TOF data—including microenvironmental cell type/state prevalence and spatial characteristics—to train a classifier for predicting DCIS recurrence, achieving notable predictive performance. Based on the gastric single-cell transcriptome atlas established in our previous work, we identified a population of KLK10^+^ early GC-specific cells, and further analyses revealed the presence of these cells in certain high-risk LGD patients, suggesting their potential as risk indicators [59]. Subsequently, we applied the developed HistoCell algorithm to estimate the proportion of KLK10^+^ cells from H&E images as a risk factor and validated its utility in distinguishing LGD progressors from non-progressors within a retrospective cohort [111]. Furthermore, Becker et al. [12] demonstrated that stem-like cells constitute a malignancy continuum from normal tissue to colon cancer and that the fraction of these cells may serve as a proxy for premalignant staging. Therefore, cellular-level alterations—particularly those related to TICs during tumorigenesis—may serve as critical indicators for the development of cellular biomarkers. Currently, multi-omics profiling of TICs is primarily employed to investigate the mechanisms underlying progression [12,64]. We anticipate that future integrative analyses of TICs—including their morphological, molecular, and microenvironmental or circulatory contexts—combined with dedicated AI models capable of learning complex associations, will provide valuable guidance for the discovery of novel biomarkers and the precise assessment of PML progression risk (Fig. 3).

### Pharmacological intervention for cancer prevention

In addition to risk stratification, therapeutic intervention represents another major avenue of ongoing research in cancer prevention. Although surgical excision of PMLs is common, such invasive procedures are not recommended for many early-stage lesions, and the risk of recurrence remains considerable even after resection. Alternatively, chemoprevention holds promise for reducing the risk of malignant transformation. While several established agents or targets, such as aspirin or other selective COX-2 inhibitors like celecoxib, have been evaluated for their efficacy in preventing cancer [133], novel chemopreventive candidates are being identified through omics-driven discovery. Nakagawa et al. [134] performed a transcriptomic meta-analysis of cirrhosis and identified the lysophosphatidic acid pathway as a candidate chemopreventive target, which was subsequently validated in both in vivo and ex vivo models. Flanagan et al. [135] identified NOTUM, a secreted WNT antagonist overexpressed by Apc-mutant intestinal stem cells, as a critical driver of early clonal expansion and adenoma formation, and demonstrated that targeting NOTUM restored wild-type cell competitiveness and prevent adenoma initiation. Moreover, as aberrant epigenetic patterns are frequently observed in premalignant states, low-dose epigenetic therapies may also exert preventive effects [28].

Immunotherapy has demonstrated efficacy in the treatment of various cancers, and, as previously noted, immune evasion often primes tumors for invasion [12,47]. Accordingly, remodeling the immune microenvironment to leverage the immune system’s capacity to intercept premalignant progression, namely, immunoprevention, represents a promising approach for cancer prevention. Notably, the efficacy of the widely used anti-PD-1 therapy has been evaluated in the context of PMLs, including in oral leukoplakia [136] and DCIS [137]. Moreover, multi-omics approaches are facilitating the identification of novel immunoprevention targets. For example, leveraging deregulated immune-related expression patterns identified in cirrhosis, Moeini et al. [49] demonstrated that administration of nintedanib and aspirin/clopidogrel in fibrotic mice resulted in down-regulation of immune-related gene expression in the liver and a reduction in both the number and size of tumors. A recent study utilized spatial immune profiling to analyze 114 human LUAD and precursor lesions, identifying a critical role of TIM-3 up-regulation in precancers [138], a finding further validated by scRNA-seq in 5 distinct mouse models. The authors subsequently demonstrated that TIM-3 blockade at the precancerous stage in mice reduced tumor burden, which was associated with increased antigen presentation, enhanced T cell activation, and a higher M1/M2 macrophage ratio. This study exemplifies the utility of multi-omics analyses in identifying potential targets for cancer interception. In parallel, vaccination strategies, such as the HPV vaccine, have demonstrated the capacity to mobilize the immune system for effective cancer prevention [139].

Given that the progression from PMLs to cancer involves a complex interplay of multiple genes and pathways, single-target interventions may be inherently limited. In this context, the network pharmacology paradigm provides a systematic framework for therapeutic intervention [140–142]. Therefore, combinatorial intervention strategies, such as those exemplified by traditional Chinese medicine (TCM), may offer additional advantages for cancer prevention, which have demonstrated efficacy in preventing cancer across multiple organs, including the liver and colon [143,144]. Furthermore, computational network pharmacology methodologies that utilize network-based modeling to elucidate drug mechanisms aim to understand drug–host interactions from a holistic perspective and have been successfully applied in numerous studies addressing the treatment of PMLs [145,146]. Thus, it is anticipated that future comprehensive multi-omics analyses of PMLs could facilitate the elucidation of the “network targets” underlying tumorigenesis; meanwhile, network pharmacology approaches, as well as the AI-based high-precision and high-efficiency R&D platform established by our group, the UNIQ system (Using Network target for Intelligent and Quantitative analysis on drug actions), may facilitate the discovery or repurposing of herbs and compounds that can synergistically target these pathways to intercept cancer initiation [142,147].

### Challenge of clinical translation from PML omics study

Although multi-omics studies of PMLs have generated valuable insights for preventing tumor initiation, it must be acknowledged that their implementation remain limited in clinical practice. Beyond the aforementioned challenges, such as the rarity of TICs and the difficulty of building high-quality longitudinal clinical cohorts, substantial obstacles still hinder the translation of multi-omics data and discoveries into actionable clinical applications.

For PML risk screening, limitations in sensitivity and specificity, or uncertainty about these metrics, remain prominent. As previously mentioned, the focal nature of PMLs renders circulating analytes exceedingly scarce, leading to low signal-to-noise ratios in blood-based assays and necessitating more sensitive technologies for reliable detection [132]. In parallel, molecular or cellular risk markers derived from tissue multi-omics are often constrained by relatively small sample sizes and potential algorithmic overfitting, resulting in limited generalizability and a lack of rigorous external evaluation. Accordingly, progress depends not only on advances in measurement technologies and computational methods, but also on carefully designed prospective validation studies that can build a robust, translational evidence base [148].

Economic and accessibility considerations are also non-negligible. For instance, while multi-omics and multimodal combinations can improve accuracy, the accuracy gains may not translate into proportional improvements in clinical utility or cost-effectiveness. A more practical approach is to implement efficient sequential cascades to enhance overall efficiency and positive predictive value; for example, a broad triage model that integrates clinical and blood-based information for population-level screening, followed by a confirmatory model that integrates molecular information with digital pathology targeted for precise risk stratification.

For evaluating preventive strategies, ethical considerations are essential when interventions are intended to halt PML progression. Extensive preclinical work is required to establish mechanisms and safety, from organoid systems to in vivo mouse studies. In parallel, innovative clinical trials are warranted in high-risk PML populations, prioritizing surrogate indicators and intermediate molecular endpoints so that efficacy can be assessed without requiring the actual onset of invasive cancer [149].

## Summary and Perspective

A substantial proportion of cancers arise from PMLs, providing a crucial window for timely diagnosis and intervention in latent tumors. Multi-omics technologies, together with integrative and dynamic analytical approaches, are essential for elucidating the molecular and cellular mechanisms underlying tumorigenesis. In this review, we first systematically summarize the classification of PMLs across multiple organs, most of which represent precursors to squamous cell carcinoma or adenocarcinoma. We then review multi-omics studies of PMLs, including bulk, single-cell, and spatial omics, with particular emphasis on those elucidating tumorigenic processes, and discuss key challenges in PML multi-omics research. We further summarize computational methodologies commonly employed in premalignant research, including multi-omics integration with recent AI advances, as well as evolutionary and dynamic analyses based on transcriptomic or genomic signals. Finally, we discuss 2 major translational directions: the development of biomarkers for risk stratification of premalignant progression, and the identification of therapeutic agents to intervene in PMLs and ultimately prevent cancer, along with their translational challenges. We hope that this review will facilitate a more comprehensive understanding and investigation of PMLs in the research community.

Given that a more comprehensive multi-omics profiling is crucial for better understanding the progression from PMLs to malignancy, we highlight several directions that could further advance tumorigenesis research:

Firstly, PML samples should be profiled by a combination of more diverse omics techniques. For example, integrative analysis of single-cell ATAC-seq and RNA-seq data is instrumental in elucidating critical regulatory molecular events underlying tumorigenesis and in identifying potential upstream therapeutic targets [150]. Simultaneous acquisition of genomic and transcriptomic information is essential for the accurate identification of TICs [151]. In addition, spatial omics approaches can be expanded to enable joint spatial multi-omics profiling. A key advantage of spatial sequencing is the capacity to perform multi-omics profiling on consecutive tissue sections, such as spatial genomics and transcriptomics [152], thereby facilitating the precise localization of critical malignant foci and characterization of their molecular and cellular transitions. Moreover, rapid advances in spatial omics technologies have enabled high-precision spatial profiling at subcellular resolution [153], providing unprecedented detail for observing tumorigenesis and facilitating the precise localization of key cell populations involved in the transition from premalignant to malignant states [84]. These include sequencing-based platforms such as Visium HD, that enable whole-transcriptome coverage at relatively low depth, and imaging-based technologies such as Xenium in situ and TF-seqFISH, which capture a subset of the transcriptome. With ongoing technological innovation, it is anticipated that future sequencing platforms will integrate these advantages, thereby providing unprecedented resolution for studying tumorigenesis.

Secondly, more sophisticated computational methodologies are required to fully exploit the potential of existing massive multi-omics datasets. For instance, the emergence of foundation models trained on diverse omics modalities presents unprecedented opportunities for integrative research on PMLs [107,154]. We anticipate that constructing and fine-tuning foundation models with large-scale single-cell and spatial multi-omics data from normal, premalignant, and malignant tissues will provide novel insights into premalignant biology. Meanwhile, the development of multimodal approaches for inferring the dynamic evolution of premalignant clones, such as leveraging high-resolution spatial transcriptomics and inferred genomic signals, will enable more accurate reconstruction of tumorigenic trajectories and precise spatiotemporal mapping of cellular and molecular events during tumorigenesis, thereby facilitating the systematic elucidation of the full landscape of tumorigenesis.

Finally, multi-omics research on PMLs should be more closely incorporated with clinical practice, to more accurately distinguish PMLs that necessitate intervention from those likely to remain indolent. On one hand, utilizing archived samples in retrospective cohort studies to identify high-risk premalignant cases, as well as designing large-scale, long-term prospective follow-up cohorts and diversified clinical sampling strategies, can provide invaluable resources for elucidating the mechanisms of tumorigenesis [155]. These outcome-annotated clinical resources facilitate the development of robust biomarkers for risk stratification and the identification of effective therapeutic targets to prevent or halt malignant transformation. These resources also support the development of multimodal AI systems that integrate electronic medical records, histopathology, and multi-omics data to enable precise prediction of progression risk and therapeutic efficacy. On the other hand, well-designed prospective clinical trials are essential for validating candidate risk biomarkers and preventive targets. We anticipate that advances across these fronts will ultimately enable the early identification and intervention of high-risk individuals, before malignant transformation occurs.
